# The Cytotoxicity of Tungsten Ions Derived from Nanoparticles Correlates with Pulmonary Toxicity

**DOI:** 10.3390/toxics11060528

**Published:** 2023-06-13

**Authors:** Jun Yao, Pengfei Zhou, Xin Zhang, Beilei Yuan, Yong Pan, Juncheng Jiang

**Affiliations:** 1 College of Safety Science and Engineering, Nanjing Tech University, Nanjing 211816, China; 2 Jiangsu Key Laboratory of Hazardous Chemicals Safety and Control, Nanjing Tech University, Nanjing 211816, China; 3 School of Environment and Safety Engineering, Changzhou University, Changzhou 213164, China

**Keywords:** tungsten carbide nanoparticles, epithelial cells, macrophages, cytotoxicity

## Abstract

Tungsten carbide nanoparticles (nano-WC) are prevalent in composite materials, and are attributed to their physical and chemical properties. Due to their small size, nano-WC particles can readily infiltrate biological organisms via the respiratory tract, thereby posing potential health hazards. Despite this, the studies addressing the cytotoxicity of nano-WC remain notably limited. To this purpose, the BEAS-2B and U937 cells were cultured in the presence of nano-WC. The significant cytotoxicity of nano-WC suspension was evaluated using a cellular LDH assay. To investigate the cytotoxic impact of tungsten ions (W^6+^) on cells, the ion chelator (EDTA-2Na) was used to adsorb W^6+^ from nano-WC suspension. Subsequent to this treatment, the modified nano-WC suspension was subjected to flow cytometry analysis to evaluate the rates of cellular apoptosis. According to the results, a decrease in W^6+^ could mitigate the cellular damage and enhance cell viability, which indicated that W^6+^ indeed exerted a significant cytotoxic influence on the cells. Overall, the present study provides valuable insight into the toxicological mechanisms underlying the exposure of lung cells to nano-WC, thereby reducing the environmental toxicant risk to human health.

## 1. Introduction

Recently, in the field of nanomaterials, there has been a notable trend of development and a substantial increase in research, leading to the continuous introduction of engineered nanomaterials into the market. The impact of nanoparticle exposure on occupational health has garnered significant attention within the field of occupational health and safety [1]. Among various nanomaterials, tungsten carbide nanoparticles (nano-WC) are recognized for their unique properties that contribute to the enhancement of metal hardness and stability [2,3]. Nano-WC is typically sprayed onto heavy machinery, drill bits, and saw blades, substantially enhancing their strength, durability, and wear resistance [4]. These properties, which are used for maintaining the sharpness of saw blades and drill bits in the mining and drilling industries, render nano-WC especially valued in these sectors. However, as the industrial-scale production and application of nano-WC increases, the dust generated from cutting, grinding, and polishing of WC-based materials poses significant occupational health risks to workers [5,6].

Epidemiological and toxicological studies have shown that nano-WC adversely affects respiratory and cardiovascular systems. Exposure to hard metal dust containing nano-WC is associated with an increased risk of occupational asthma and hard metal lung disease (HMLD) [7], which is characterized by difficulty breathing, reduced lung capacity, progressive lung inflammation, and pulmonary fibrosis [8,9,10,11]. Additionally, studies have found that tungsten element present in nano-WC has adverse impact on cell viability [12]. It also interferes with the voltage-gated sodium channels in neurons [13], thereby inducing cell apoptosis [6] and inflammation [14]. However, current in vitro studies on WC are limited to fish cell lines [15], and the cytotoxicity of nano-WC has not been reported.

Macrophages are utilized to clear infectious, toxic, or allergenic particles from the airways, while epithelial cells serve to protect underlying tissues from damage. However, nanoparticles (NPs), when exposed to macrophages and epithelial cells, can induce cytotoxicity due to their internalization, subsequently leading to serious lung diseases [16,17]. Reports suggested that in animal model studies, small nanoparticles (NPs) deposited in the respiratory tract easily infiltrate into epithelial and interstitial sites [18,19]. Large NPs can attenuate or inhibit the phagocytic action of macrophages in the alveoli [20]; thus, the phagocytosis of NPs by epithelial cells or macrophages may depend on their size. In vitro experiments have shown that NPs loaded with 2 μg WC can cause significant toxicity to alveolar macrophages with 24 h of exposure at concentrations ranging from 50 to 1667 μg/mL. Furthermore, mouse peritoneal macrophages exposed to between 50 and 300 μg/mL NPs with 2 to 4 μg WC showed signs of toxicity within 6 h of exposure [21]. In a recent study [22], a co-culture model of lung epithelial cells and macrophages was established to simulate the microenvironment of the lung, and examine the toxic and inflammatory effects of tungsten carbide cobalt (WCCo) nanoparticles (NPs). Furthermore, mechanisms underlying lung toxicity due to NPs need to be investigated.

Studies show that the slow release of ions in vivo or in cells is the main cause of cytotoxicity. Song et al. [23] found that dissolved Zn^2+^ plays a major role in mediating the toxic effect of ZnO particles by generating large amounts of reactive oxygen species (ROS) in the cells. Yosuke et al. [24] evaluated the effects of indium tin oxide nanoparticles (ITO NPs), indium chloride (InCl_3_) and tin chloride (SnCl_3_) on A549 cells, revealing that the accumulation of indium ions in cells induces oxidative stress, proinflammatory response and DNA damage. In addition, in vitro experiments have also confirmed that indium ions released from ITO particles are the primary source of cytotoxicity and genotoxicity [25,26,27,28]. In vitro studies on mammalian cells have shown that the effects on different organs and developmental physiology are dose-dependent [29]. However, the underlying mechanism of nano-WC-induced cytotoxicity is still unrevealed.

In this study, we evaluated the solubility and cytotoxic effects of nano-WC on macrophage and lung epithelial cells. We investigated the apoptosis of U937 macrophage and BEAS-2B epithelial caused by a nano-WC suspension. Furthermore, the metal ion chelator of EDTA-2Na was used to reduce W^6+^ from nano-WC. Subsequently, the rates of cellular apoptosis was measured before or after chelation treatment, thereby validating the effect of W^6+^ on cytotoxicity. Our findings provide a reference for subsequent in vivo studies on the mechanisms of nano-WC toxicity.

## 2. Materials and Methods

### 2.1. Materials

Nano-WC (purity ≥ 99.99%) were obtained from Boxin Wear-Resisting Alloy Material Co., Ltd. (Xingtai, China). The hydroclynamic size of nano-WC is around 60 nm as shown in Figure S1. In addition, the zeta potential test indicates that the nano-WC is electrically negative at test conditions (Figure S2). They were tested with a Zetasizer Nano ZS ZEN3600 (Malvern, Worcestershire, UK) electrokinetic analyzer. After weighing and autoclaving at 121 °C and 0.12 MPa for 30 min, a nano-WC suspension (200 μg/mL) was prepared in high glucose DMEM (Cytiva, Shanghai, China, for culturing BEAS-2B cells) and RPMI-1640 (ThermoFisher, Shanghai, China, for culturing U937 cells) media, and stored at 4 °C. Before each experiment, the suspension was dispersed in a sonicator for 15 min and then diluted to the required concentration.

### 2.2. Cell Lines

The human lung epithelial cell line BEAS-2B and the human macrophage cell line U937 were purchased from American Type Culture Collection (ATCC), Shanghai Cell Bank. The cells were cultured in DMEM (Cytiva, Shanghai, China) and RPMI-1640 (ThermoFisher, Shanghai, China) medium supplemented with 10% (*v*/*v*) fetal bovine serum (Gibco, Shanghai, China) and 1% (*v*/*v*) penicillin–streptomycin solution (ThermoFisher, Shanghai, China) at 37 °C in an incubator with 5% CO_2_, respectively.

### 2.3. Cell Culture and Measurement of Tungsten Ion Release

BEAS-2B and U937 cells were cultured for 12 h in 24-well plates (1 × 10^5^ cells per well), and washed thrice with PBS to remove non-adherent cells. The cells were then treated with nano-WC of 200 μg/mL for 1, 2, 6, 12, and 24 h. The supernatants were then centrifuged thrice, and the concentration of W^6+^ was measured by inductively coupled plasma mass spectrometry (ICP-MS). A Thermo Electron X Series X7 quadrupole ICP-MS (ELEMENT 2, Shanghai, China) was used. Samples were introduced into the ICP torch using a quartz C-type nebulizer (OpalMist, Beijing, China) and the impact bead spray chamber was cooled to 2 °C. In order to obtain the maximum sensitivity of tungsten, ICP-MS is generally tuned to 1600–1700 V. The retention time of each sample is 10 s, and the concentration of the target element of the measured sample is less than 1 ppm. After the measurement, the system was cleaned with 2–4% dilute nitric acid for 5–10 s, and the concentration and relative standard deviation (RSD) were checked and recorded.

### 2.4. Transmission Electron Microscopy

BEAS-2B and U937 cells were put in 24-well plates at a density of 1 × 10^5^ cells/well. 50 μg/mL nano-WC suspension was treated as test group. Each group of cells were fixed with 2.5% glutaraldehyde in 0.1 mol/L phosphate buffer (pH = 7.2) for 1 h at ice temperature and post-fixed with 1% OsO_4_ in the same buffer for 2 h at room temperature. After fixation, cells were dehydrated with acetone (30%, 50%, 70%, 80% and 90%) and embedded in Spurr resin. Thin sections of 50 nm were cut at the ultramicrotome (RMC POWERTOME XL, RMC, USA) and deposited on 200 mesh copper grids. Fixed samples were stained with uranyl acetate and lead citrate at room temperature for 10 and 12 min, respectively. The cell membrane, chromatin, nucleus, and intracellular particle size distribution were characterized using a 200 kV field emission transmission electron microscope (JEM-2100F) by Japan JEOL.

### 2.5. Cytotoxicity Analysis

LDH release was analyzed by using the LDH Cytotoxicity Assay Kit (C0016, Beyotime, Shanghai, China) in accordance with the manufacturer’s instructions. Briefly, the cells were put in 96-well culture plates at a density of 1 × 10^5^ cells/well and incubated for 12 h. Background blank wells, sample control wells, sample maximum enzyme activity control wells and sample wells were set up. We added 200 μL supernatant to each sample well for another 1 h incubation. Until 1 h before detection, 13 μL of LDH release reagent was added to the “sample maximum enzyme activity control well”. Then, 60 μL of LDH detection reagent was added and incubated for 30 min. The supernatant medium was placed in 96-well culture plates, and the absorbance at 490 nm was measured by a microplate reader (EPOCH2, BioTek, Vermont, USA).Mortality is calculated by the following formula:

(1) $$M=\frac{A_{treated}-A_{control}}{A_{activecontrol}-A_{control}}\times 100\%$$

where *M* is cellular mortality; *A_treated_* is the absorbance of samples exposed to nano-WC; *A_control_* is absorbance of untreated control wells; and *A_active control_* is absorbance of maximum enzyme activity of cells.

To validate the cytotoxicity derived from W^6+^ rather than nano-WC, we conducted cell viability experiments using EDTA-2Na (50 μg/mL) adsorbed W^6+^ as a comparison. BEAS-2B and U937 cells were seeded into 96-well plates at a density of 10^5^ cells/well and cultured for 12 h. They were then divided into control, WC, and WC + EDTA-2Na groups, and exposed to nano-WC at a concentration of 200 μg/mL. The Annexin V- Phycoerythrin (PE) 7- amino-actinomycin D (7-AAD) apoptosis detection kit (cat. no. KGA1017; KeyGEN Bio TECH, Nanjing, China) was used to detect cell death according to the manufacturer’s protocol. Cells were resuspended in 100 μL of staining solution at a concentration of 1 × 10^5^ cells/100 μL and incubated in the dark at ambient temperature for 10 min. They were then added to 400 μL of binding buffer. A flow cytometer (Beckman FC-500, Brea, CA, USA) was employed to determine the excitation wavelength at 488 nm. The excitation wavelength at 578 or 647 nm was used to detect PE or 7-AAD fluorescence for cell apoptosis, respectively. The cells can be divided into three subgroups. The viable cells only showed very low intensity of background fluorescence, the early apoptotic cells only showed strong orange-red fluorescence, and the late apoptotic cells showed the double staining of the orange-red and red fluorescence. Samples (*n* = 10) were randomly selected from each group, and the apoptotic rate was calculated as the percentage of early apoptotic cells or late apoptotic cells.

### 2.6. Statistical Analysis

Origin 8.0 was used for all statistical analyses. The data were presented as mean ± standard deviation (SD) and the comparison between the two groups was performed using the Student’s *t*-test (for parametric data). *p* values < 0.05 were considered statistically different.

## 3. Results

### 3.1. Decrease in the Viability of Lung Epithelial Cells and Macrophages by Nano-WC

**Figure 1:**
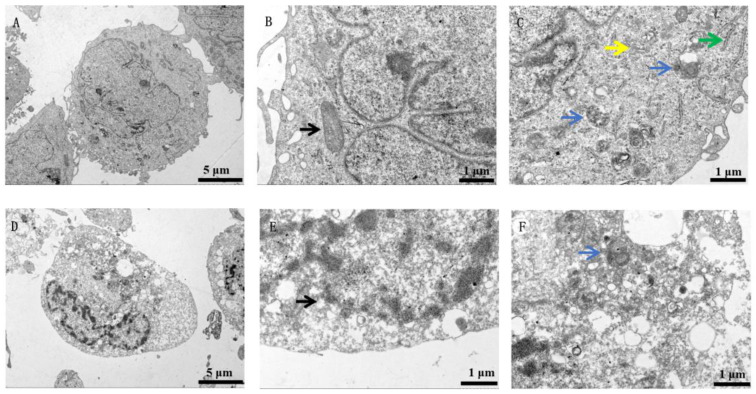
TEM images of BEAS-2B cells in the control group. (**A**) The cell membrane and the nuclear membrane are intact. (**B**) Mitochondria are clear and intact (black arrows) with clear ridges. (**C**) The lysosomes (blue arrows), Golgi apparatus (yellow arrows), and endoplasmic reticulum (green arrows) are clear and intact. Sonicated WC solution was added to BEAS-2B cells and incubated for 24 h. TEM images of BEAS-2B cells exposed to nano-WC. (**D**) The cytosol and nucleus with a nuclear membrane are largely intact. The nucleoplasm is condensed around the nuclear membrane, and there are numerous intracellular vacuoles. (**E**) Mitochondria (black arrows) are poorly defined. (**F**) There are numerous intracellular vacuoles and the lysosomes (blue arrows) are poorly defined.

**Figure 2:**
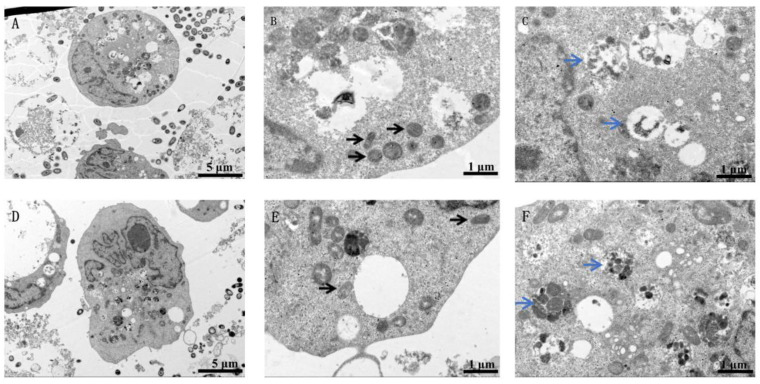
TEM images of U937 cells in the control group. (**A**) The overall cell structure is intact. The mitochondria ((**B**); black arrows) and lysosomes ((**C**); blue arrows) are intact. Sonicated WC solution was added to U937 cells and incubated for 24 h. TEM images of U937 cells exposed to nano-WC. (**D**) The overall cell structure is essentially intact, with an irregular nuclear membrane and a solid nucleolus. (**E**) There are large vacuoles and multiple mitochondria (black arrows) in the cytoplasm. (**F**) The lysosomes (blue arrows) are segmented internally.

Compared to the untreated BEAS-2B cells (Figure 1A–C), those treated with nano-WC particles show indistinct organelles, indistinct mitochondrial profiles and lysosomes, numerous vesicles, and a condensed nucleoplasm around the nuclear membrane (Figure 1E,F), although the overall cell structure is intact (Figure 1D). Similarly, the control U937 cells have an intact cell membrane and nuclear membrane, along with multiple mitochondria (Figure 2A–C). While the overall structure of these cells is unaffected by nano-WC (Figure 2D), they exhibit an irregular nuclear membrane and a solid nucleolus, large vacuoles, multiple mitochondria (Figure 2E), and segment extracellular lysosomes (black arrows; Figure 2F).

### 3.2. Release of Tungsten Ions in Cells Exposed to Nano-WC

**Figure 3:**
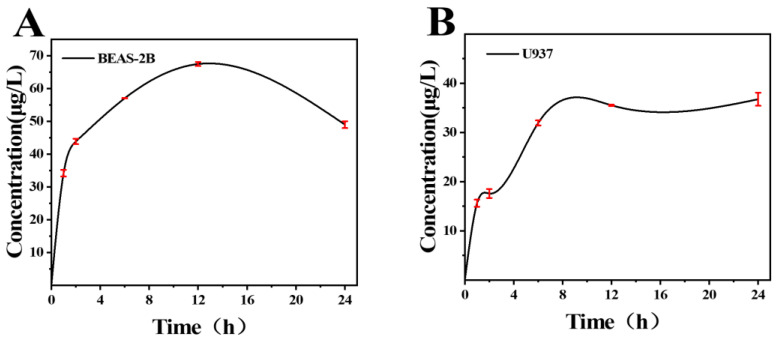
Temporal dissolution curves of nano-WC in BEAS-2B and U937 cells. (**A**) W^6+^ concentration in the supernatants of BEAS-2B treated with 200 μg/mL nano-WC for 0, 1, 2, 6, 12, and 24 h. (**B**) W^6+^ concentration in the supernatants of U937 treated with 200 μg/mL nano-WC for 0, 1, 2, 6, 12, and 24 h.

Studies show that the cytotoxicity of metal oxide NPs is mediated by the release of free metal ions [23,30]. Therefore, we hypothesize that the release of W^6+^ from nano-WC is the source of toxicity. To this end, lung epithelial cells and macrophages were treated with 200 μg/mL nano-WC, and the W^6+^ levels in the supernatant were measured by ICP-MS after centrifugation [31]. As shown in Figure 3A,B, at the initial time of 0 h prior to the treatment of cells, the detected W^6+^ concentration in the supernatant with only nano-WC was 0, implying that nano-WC does not spontaneously dissolve in the absence of cells. Within 12 h, the changes in the W^6+^ concentration released from nano-WC exposed to both cell types were generally consistent. After 12 h, the W^6+^ concentration decreased but remained high in the supernatant of BEAS-2B cells, while the W^6+^ concentration in the supernatant of U937 cells was relatively stable. Generally speaking, W^6+^ concentrations in the supernatants of both cell types increased significantly after 24 h of incubation.

### 3.3. Mitigating the Cytotoxicity of Nano-WC by Chelation of W^6+^

**Figure 4:**
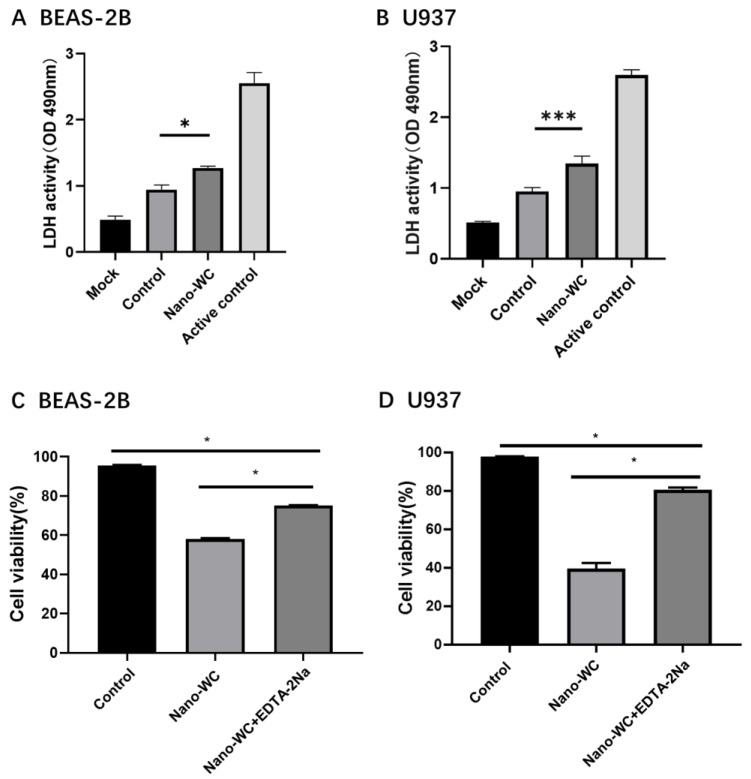
LDH activity of BEAS-2B cells (**A**) and U937 cells (**B**) treated with the condition medium of nano-WC-treated cells. LDH activity of BEAS-2B cells (**C**) and U937 cells (**D**) treated with the condition medium with or without EDTA-2Na. The data on the figures represent mean ± SD. * *p* < 0.05 and *** *p* < 0.001 vs. control group.

To detect the toxic effect of tungsten ion concentration in supernatant on cells, lactate dehydrogenase (LDH) levels were measured (Figure 4A,B). The cells in the nano-WC groups release significantly higher amounts of LDH compared to the control groups, and the mortality rates of BEAS-2B and U937 cells are 20% and 24%, respectively. The results showed that tungsten ions in the supernatant could also induce the release of LDH in BEAS-2B cells and U937 cells, leading to apoptosis.

**Figure 5:**
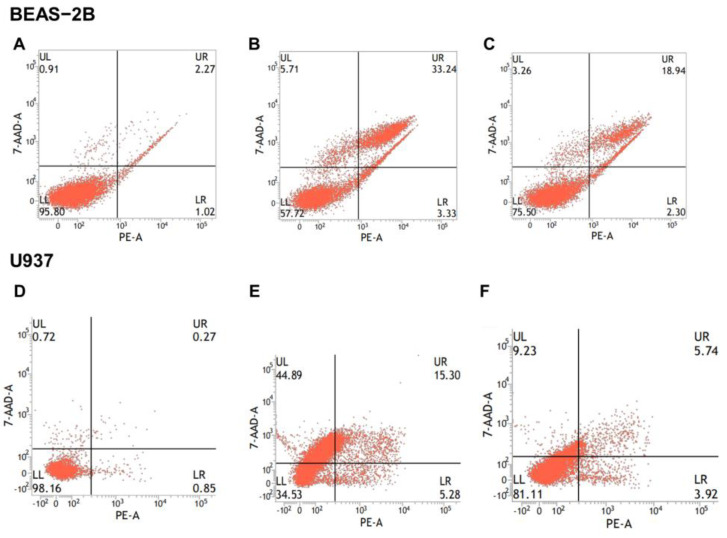
Flow cytometry measurements of BEAS-2B cells and U937 cells in the control (**A**,**D**), nano-WC (**B**,**E**), and nano-WC + EDTA-2Na (**C**,**F**) groups. Apoptosis rates were calculated based on Figure 5.

To investigate the cytotoxic impact of tungsten ions (W^6+^) on cells, EDTA-2Na was used to chelate W^6+^ from nano-WC suspension. Subsequent to this treatment, the modified nano-WC suspension was subjected to flow cytometry analysis (Figure 5) to evaluate the rates of cellular apoptosis. The apoptosis rate of BEAS-2B cells in the nano-WC group was 36.57%; the apoptosis rate of EDTA-2Na group was 21.24%. The apoptosis rate of U937 cells in the nano-WC group was 20.58%, while that in EDTA-2Na group was 9.62%. The viability of cells treated with EDTA-2Na and nano-WC is significantly higher than that of cells treated with nano-WC alone (*p* < 0.05; Figure 4C,D). The result shows that the addition of EDTA-2Na will reduce the W^6+^ concentration, and thus toxicity will be reduced.

## 4. Discussion

The nucleus controls cellular functions and the inheritance of genetic material [32,33], and the aggregation and condensation of chromatin in the nucleus is an indicator of irreversible damage [34]. We found that nano-WC exposure led to significant chromatin condensation in human lung epithelial cells, along with loss of organelle integrity and a massive increase in the number of vesicles, which could be the cellular basis of the toxic effects of nano-WC. Recent studies have shown that lysosomal damage can trigger apoptosis [35,36]. In addition, phagocytosis of inhaled silica or asbestos dust by lung macrophages leads to lysosomal rupture, which releases hydrolytic enzymes and increases tissue fibrosis [37]. Consistent with this, the U937 cells treated with nano-WC particles showed split extracellular lysosomes.

A number of studies have proven that free metal ions released from metal oxide NPs are the primary factor for cytotoxicity [23,27,28,30]. Singh et al. [38] demonstrated that phagocytosed Ag NPs are degraded inside the cells and release Ag ions, which interfere with normal mitochondrial functions and induce apoptosis. Likewise, we detected free W^6+^ in the supernatant of the cell lines, and the release of tungsten ions is increased with increased time during the first 12 h (Figure 3A,B). Overall, tungsten ion concentrations in the supernatants of both cell types increased significantly after 24 h of incubation, and these data suggest that macrophages and epithelial cells can rapidly (within 24 h) dissolve nano-WC into W^6+^, which are then released by dying cells into the surrounding medium.

LDH is an important cell metabolism enzyme in body tissues, which can better reflect cell proliferation and metabolism [39]. The LDH level of healthy tissue cells is generally low, and when tissue cells are damaged, the LDH level will rise. Indeed, the increase in LDH activity confirms that the tungsten ions in the supernatant could also induce the release of LDH in both cells, leading to apoptosis. This indicates that after nano-WC enters the body, BEAS-2B cells and U937 cells had similar outcomes as target cells, providing an experimental basis for subsequent animal experiments to select target organs. Furthermore, to validate the cytotoxicity derived from W^6+^ rather than nano-WC, EDTA-2Na was used to adsorb W^6+^. EDTA-2Na can effectively prevent metal ions from acting by encapsulating metal ions into the chelating agent through its strong binding with metal ions [40,41,42], which effectively avoid the induction of free radicals/reactive oxygen species (ROS) causing oxidative stress by chelating the free W^6+^ [43].These results support the hypothesis that nano-WC particles are phagocytosed by macrophages, degraded in the acidic lysosomes, and release W^6+^ that triggers apoptosis. Nanoparticle solubilization results in a local spike in ionic tungsten, which damages and permeabilizes the lysosome, causing the contents (along with the ionic tungsten) to leak out into the cytoplasm, which kills the cell [28]. Furthermore, free W^6+^ is significantly more toxic than nano-WC and can easily target neighboring macrophages or lung epithelial cells [28,44,45]. This suggests that the partial cause of cytotoxicity is W^6+^ released from nano-WC, and W^6+^ can indeed cause cell damage in the absence of nano-WC.

Moreover, apart from the toxic effect of NPs on the cell level, the effect of pulmonary surfactant on NPs should not be ignored. For different kinds of NPs, many studies have demonstrated that the pulmonary surfactant will promote or reduce the toxicity of NPs [46,47]. Therefore, the interaction of nano-WC and pulmonary surfactant needs further intensive investigation.

## 5. Conclusions

The mechanisms underlying lung toxicity due to nano-WC were investigated. U937 macrophages and BEAS-2B epithelial cells were cultured and then exposed to nano-WC. By TEM, it was found that human lung epithelial cells showed obvious chromatin condensation, loss of organelle integrity, and a large increase in the number of vesicles, as well as the division of extracellular lysosomes in U937 cells, confirming obvious cell damage. The cytotoxicity of nano-WC suspension was proven through a cellular LDH assay. Furthermore, a decrease in W^6+^ by the ion chelator (EDTA-2Na) reduces cytotoxicity, which indicates that the partial cause of cytotoxicity is W^6+^ released from nano-WC. Our findings provide new insights into the mechanisms underlying toxicity of nano-WC on the lung epithelium, and possible strategies for therapeutic intervention.

## Data Availability

The data that support the findings of this study are available from the corresponding author, (Y.P.), upon reasonable request.

## Supplementary Materials

The following supporting information can be downloaded at: https://www.mdpi.com/article/10.3390/toxics11060528/s1, Figure S1: Diameter distribution of nano-WC suspension; Figure S2: The distribution of zeta potential as a function of nano-WC concentration.
