# Supplementary material for: The Cytotoxicity of Tungsten Ions Derived from Nanoparticles Correlates with Pulmonary Toxicity
Source: Toxics. 2023 Jun 13;11(6):528. doi: 10.3390/toxics11060528 (PMC10302912; doi:10.3390/toxics11060528)
Supplement: Supplementary file 1 [file toxics-11-00528-s001.zip › toxics-2422397-supplementary.pdf]

Supplementary Material

For

The Release of Tungsten Ions Derived from Tungsten Carbide Nanoparticles Contributes to Cytotoxicity

Jun Yao[a], *Pengfei Zhou*[a], *Xin Zhang*[a], *Beilei Yuan*[a,b,*], *Yong Pan*[a,b,*], *Juncheng Jiang*[b,c,*]

[a] College of Safety Science and Engineering, Nanjing Tech University, Nanjing 211816, Jiangsu, P. R. China

[b] Jiangsu Key Laboratory of Hazardous Chemicals Safety and Control, Nanjing Tech University, Nanjing 211816, Jiangsu, P. R. China

[c] School of Environment & Safety Engineering, Changzhou University, Changzhou 213164, Jiangsu, P. R. China

* Corresponding author. Tel.: +86-25- 83239976;

E-mail: yuanbeilei@163.com (B.L. Yuan); yongpan@njtech.edu.cn (Y. Pan); jcjiang_njtech@163.com (J.C. Jiang)

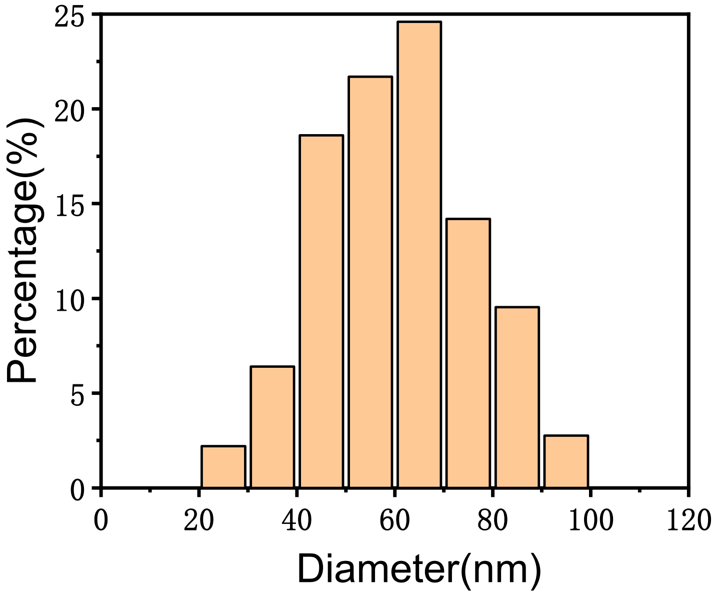

Fig. S1. Diameter distribution of nano-WC suspension.

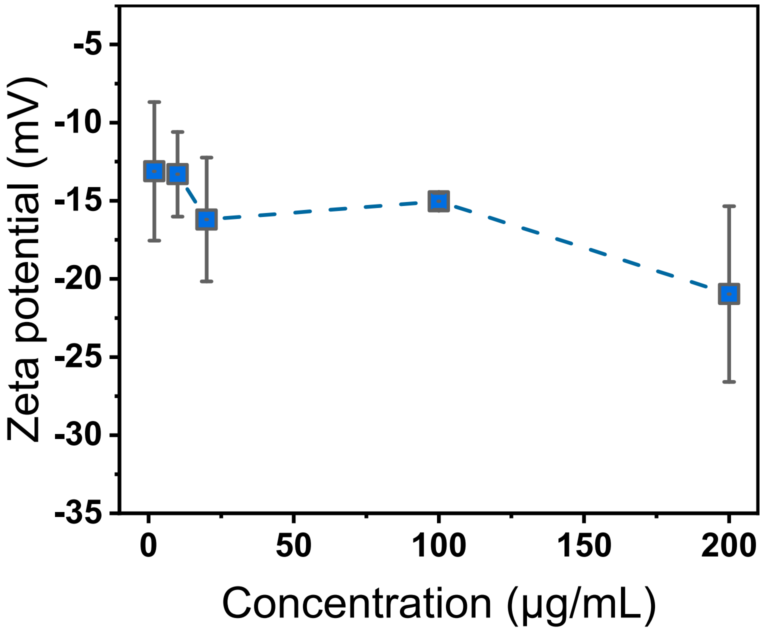

Fig. S2. The distribution of zeta potential as a function of nano-WC concentration.
